# Quantifying spatial CXCL9 distribution with image analysis predicts improved prognosis of triple-negative breast cancer

**DOI:** 10.3389/fgene.2024.1421573

**Published:** 2024-06-18

**Authors:** Xi Cao, Yu Song, Huanwen Wu, Xinyu Ren, Qiang Sun, Zhiyong Liang

**Affiliations:** ^1^ Department of Breast Surgery, Peking Union Medical College Hospital, Peking Union Medical College, Chinese Academy of Medical Sciences, Beijing, China; ^2^ Department of Pathology, Peking Union Medical College Hospital, Peking Union Medical College, Chinese Academy of Medical Sciences, Beijing, China

**Keywords:** CXCL9, triple-negative breast cancer, computational image analysis, prognosis, immune checkpoint

## Abstract

**Background:** The C-X-C motif chemokine ligand 9 (CXCL9) plays a pivotal role in tumor immunity by recruiting and activating immune cells. However, the relationship between CXCL9 expression and prognosis in triple-negative breast cancer (TNBC) is unclear.

**Methods:** We investigated CXCL9 mRNA expression, clinicopathological features, and prognosis in TNBC patients. We also used computational image analysis to quantify and assess the distribution of CXCL9 protein in the tumor core (TC) and invasive margin (IM).

**Results:** CXCL9 mRNA expression was significantly higher in TNBC tumors compared to normal tissue (*p* < 0.001) and was associated with smaller tumors (*p* = 0.022) and earlier stages (*p* = 0.033). High CXCL9 mRNA expression was correlated with improved overall survival (OS) in three independent cohorts (all *p* < 0.05). In a separate analysis, low CXCL9 protein expression was associated with increased lymph node metastasis (*p* = 0.018 and *p* = 0.036). High CXCL9 protein expression in the TC, IM, or both was associated with prolonged OS (all *p* < 0.001).

**Conclusion:** High CXCL9 expression, at both the mRNA and protein levels, is associated with improved prognosis in TNBC patients. CXCL9 expression in the TC and/or IM may be an independent prognostic factor.

## 1 Introduction

Breast cancer is the most common cancer threatening women’s health (Cao et al., 2021; Siegel et al., 2022). Triple-negative breast cancer (TNBC), characterized by the loss of expression of the estrogen receptor (ER), progesterone receptor (PR), and human epidermal growth factor 2 (HER2), is the most aggressive subtype, with early recurrence and unfavorable prognosis (Guo et al., 2023; Lee, 2023). Owing to the lack of targets for anti-HER2 and endocrine therapies, the major adjuvant treatment options for TNBC are still limited to traditional chemotherapy and radiotherapy. Moreover, the mutation rate of TNBC is significantly higher than that of other breast cancer subtypes and approximately 13.3 times that of normal cells, making it highly immunogenic (Hadi et al., 2020; McCart Reed et al., 2021). Furthermore, the tumors microenvironment of TNBC has a higher number of tumors-infiltrating immune cells than other subtypes of breast cancer (Denkert et al., 2018; Sun et al., 2020). Results from neoadjuvant and metastatic TNBC investigations suggest that it is more likely to benefit from immunotherapy based on programmed cell death ligand 1(PD-L1) inhibitors or programmed cell death (PD-1) inhibitors combined with chemotherapy (Schmid et al., 2020; Deutschmann et al., 2022). Previous studies have also demonstrated the different and distinct microenvironments related to the effect of immune checkpoint blockade (ICB) in TNBC (Xiao et al., 2019). “Hot tumors” should be treated with immunotherapy.

Chemokines are secreted proteins that mediate the transport of immune cells and are recruited into the tumors microenvironment (TME) (Dastmalchi et al., 2019; Xun et al., 2020). Chemokines play an important role in elevating immune cell infiltration, promoting “inflamed” TME, and stimulating anti-tumors immunity and responses to ICB (Vilgelm and Richmond, 2019). Chemokines are divided into CXC, CC, CX3C and C families according to their structures (Susek et al., 2018; Xu et al., 2018). They can also be divided into two subtypes, homeostatic and inflammatory, according to their functions (Nagarsheth et al., 2017). CXCL9 is a CXCR3 ligand. It recruits cytotoxic lymphocytes (CTLs), natural killer (NK) cells, NKT cells, and macrophages into the TME and promotes naïve T cell differentiation into T-helper 1(Th1) cells which respond to interferon-γ (IFN-γ) (Nagarsheth et al., 2017; Finger et al., 2019). CXCL9 within murine tumors and the plasma could be an indicator for predicting treatment response to ICB, and it could also promote ICB-based anti-PD-1 inhibition (Chow et al., 2019). “Hot tumors”, which mostly have high levels and locally enriched expression of CXCL9, are closely related to inflammation and anti-tumor reactivities (Hoch et al., 2022). IFN-γ-inducible CXCL9 is related to the activation of Th1 immunity, prolonged survival, and better responses to chemotherapy and immunotherapy (Dangaj et al., 2019). These results revealed a synergistic relationship between CXCL9 and ICB. To date, there have been limited studies on CXCL9 and breast cancer. Previous studies have demonstrated that high levels of CXCL9 mRNA are associated with prolonged survival in patients with breast cancer (Li et al., 2020), particularly the TNBC subtype (Razis et al., 2020; Lv et al., 2021).

In this study, we explored the correlation between CXCL9 mRNA expression levels, clinical features, survival and TNBC based on our cohort and public datasets. We further explored CXCL9 localization and expression in the central area and the invasive front of TNBC through computational imaging techniques, and further analyzed the relationship between the localization and expression of CXCL9 and survival outcomes in the TNBC.

## 2 Materials and methods

### 2.1 Patient characteristics

In total, 239 patients with stage I - III TNBC who had undergone radical surgery at the Department of Breast Surgery, Peking Union Medical College Hospital (PUMCH) between 2009 and 2014 was recruited. These patients were enrolled in the PUMCH TNBC cohort 1 (*n* = 187) and the PUMCH TNBC cohort 2 (*n* = 69), with 17 patients were enrolled in both cohorts. Patients had received non-therapy before surgery. The patients received standardized treatment, such as chemotherapy and radiotherapy, and were followed up regularly after surgery. This study conformed to the principles of the Declaration of Helsinki and was approved by the Ethical Review Board of PUMCH. Informed consent was obtained from all participating patients. Finally, a total of 187 patients were enrolled in the PUMCH cohort 1 with median follow-up time of 84 months and were further analyzed with CXCL9 mRNA expression. A total of 69 patients were enrolled in the PUMCH cohort 2 with median follow-up time of 92 months and were further analyzed for location and quantity of CXCL9 expression and survival.

### 2.2 Detection of CXCL9 mRNA expression

CXCL9 mRNA were detected in 187 patients. Total RNA was extracted from formalin-fixed paraffin-embedded (FFPE) breast cancer tissue for each breast cancer specimens using an RNA Storm RNA extraction kit (CD201, CELLDATA, Fremont, CA, USA). Gene expression was detected using RNA-seq as described previously (Yang et al., 2018). Briefly, a customized Illumina TruSeq Target RNA Expression Kit was used to build libraries of CXCL9 and six housekeeping genes (GAPDH, GUSB, MRPL19, PSMC4, SF3A1, and TFRC), each of which was loaded onto the iSeq100 for sequencing according to the manufacturer’s protocols. Illumina Casava1.7 software was used for base calling, and sequencing data were demultiplexed using Illumina bcl2fastq2 software to generate one FASTQ file per sample. Raw counts of all samples were normalized by the size of the transcripts and library, and then, calculated as counts per million (CPM) for each sample as a gene expression matrix using R package edge R from Bioconductor. Log-based 2 values were transformed into the present expression values. The expression of CXCL9 was normalized to the average of the six housekeeping genes for each sample. Gene expression data were further median-centered and standardized for analysis.

### 2.3 Gene analysis of the TNBC cohorts in the GEO and TCGA dataset

Datesets of GSE76250 based on GPL17586 platform and GSE58812 based on GPL570 platform were obtained from the Gene Expression Omnibus (GEO) (https://www.ncbi.nlm.nih.gov/geo/). The GSE76250 contained 33 normal breast tissue samples and 165 TNBC samples, in which 154 TNBC samples contained complete clinical pathological information (age, menopause status, tumor size, positive lymph nodes, ki67 index and tumor grades) were used for analysis. The GSE58812 contained 107 TNBC samples with complete follow-up information and were used for analysis.

We downloaded the gene-level RNA-seq expression data and clinicopathological information for breast invasive carcinoma (BRCA, PanCancer Atlas) from The Cancer Genome Atlas (TCGA) dataset (http://www.cbioportal.org/) and screened TNBC patients. The inclusion criteria were as follows. (1) Female patients diagnosed with stage I-III breast cancer with complete clinical information. (2) Immunohistochemical (IHC) staining demonstrated IHC-ER-negative and IHC-PR-negative. (3) Tumour determined as IHC-HER2-negative and fluorescence *in situ* hybridisation (FISH) also negative or not available, or (4) IHC-HER2-equivocal, unavailable, or indeterminate but FISH negative, or (5) IHC-HER2-positive but FISH negative. Finally, 156 patients were enrolled in the TCGA TNBC cohort.

### 2.4 CXCL9 immunohistochemical staining and computational image analysis

CXCL9 protein and other 6 marker were stained in 69 patients. Eight continuous slides of each formalin-fixed paraffin-embedded sample were obtained and immunohistochemically stained for CXCL9 (AB-9720, Abcam), CD3 (clone LN10, Leica), CD4 (clone 4B12, Leica), CD8 (clone 4B11, Leica), CD19 (clone BP6046), CD163 (clone 10D6, Leica), cytokeratin (clone AE1/AE3, Leica), and haematoxylin-eosin (H&E) according to the manufacturer’s protocol using a Leica stainer (Leica Biosystems, Germany).

**FIGURE 1:**
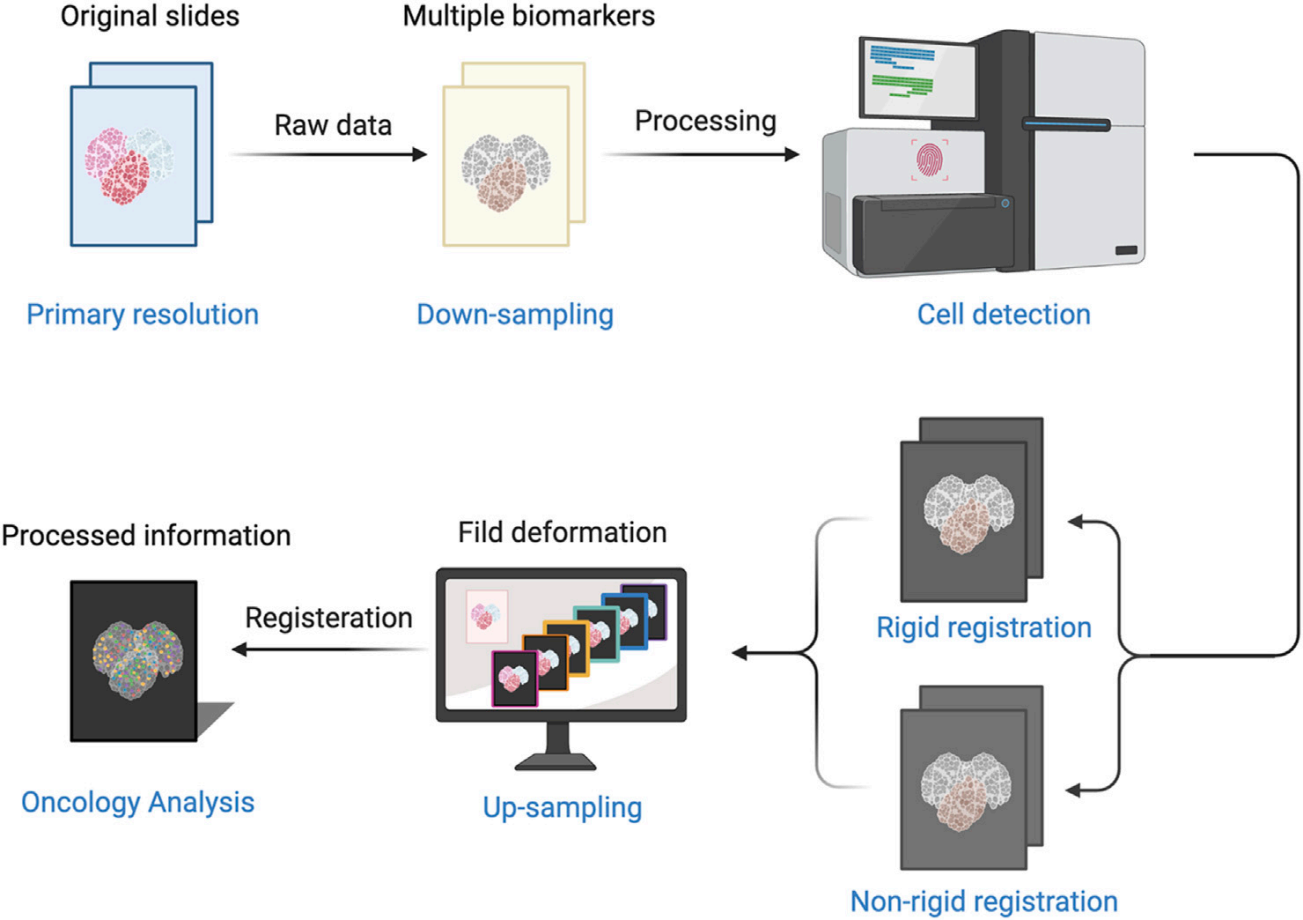
The process of computer-aided image includes image registration, annotation, and quantification.

Computational image processing included image registration, annotation, and quantification (Figure 1), with details were described in our previous study (Ren et al., 2023). First, all slides were scanned with the KF-pro-400 scanner (Ningbo, China) and converted into digital images (×40 magnification, 0.2μm/pixel), under consistent parameter settings. The scanned microscopic images were then imported as digital files and registered and aligned to the fourth image. Images were down-sampled to 500 × 500 thumbnails and aligned subsequently through shifts, rotations, and non-rigid deformations, then up-sampled to the original size using Python scripts. The tumors core (TC) and invasive front (IF) were annotated with QuPath by an experienced pathologist, while the invasive margin (IM) was defined as the region within a 100 µm extension of IF. QuPath was also used to perform cell segmentation and quantification. CXCL9 quantification mainly involved spatial analysis. The spatial distribution of CXCL9 expression was evaluated as density and percentage, respectively, in different areas including TC, IM, and the whole tumors area. The CXCL9 positive density was defined as the number of positive cells per mm2. The CXCL9 positive percentage was defined as the proportion of positive cells in each region. The above interpretation methods were also applied to the interpretation of CD3, CD4, CD8, CD19 and CD163.

### 2.5 Statistical analysis

Categorical variables were compared using the Chi-squared test. Mann-Whitney U tests were used to analyze the significance of differences between the two groups. Spearman’s correlation test analyzed the association between the expression of CD3, CD4, CD8, CD19, CD163 and CXCL9 in TC and/or IM of TNBC according to density and percentage. Receiver operating curve (ROC) analysis was used to determine the best cut-off value for each measurement. Survival analyses including disease-free survival (DFS) metastasis-free survival (MFS) and overall survival (OS) were plotted using the Kaplan-Meier method and compared with log-rank tests. Uni- and multivariate analyses were performed using Cox regression models to determine the independent prognostic factors in the cohorts. A two-sided *p* < 0.05 was considered statistically significant. SPSS (version 21.0, IBM Corp., Armonk, NY, USA) was used for statistical analysis and GraphPad Prism v9.0.2 was used for plotting.

## 3 Results

### 3.1 CXCL9 mRNA expression level and clinical features in TNBC cohorts

In the GSE76250 TNBC cohort, 64.3% were older than 50 years, 63% had tumors larger than 2 cm, 44.2% of patients had lymph node metastasis and 20.2% had more than 4 lymph nodes involved. According to the AJCC, 76.6% of patients were diagnosed with stage II-III (Supplementary Table S1). This cohort was divided into high- and low-CXCL9 expression groups based on the median mRNA level. CXCL9 expression did not correlate with age (*p* = 0.130), T stage (*p* = 0.089), N stage (*p* = 0.442), TN stage (*p* = 0.477), menopause status (*p* = 0.186), ki-67(*p* = 0.581) or histologic grade (*p* = 0.927) (Supplementary Table S2).

In the TCGA TNBC cohort, the median age of the patients was 55 years. Of the patients, 67.3% were older than 50 years, 74.4% had tumors larger than 2 cm, and 4 patients had T4 tumors. Moreover, 32.7% of patients had lymph node metastasis and 12.2% had more than 4 lymph nodes involved. According to the AJCC, 82% of patients were diagnosed with stage II-III (Supplementary Table S3). CXCL9 expression did not correlate with age (*p* = 0.953), T stage (*p* = 0.150), N stage (*p* = 0.096), or tumour-node-metastasis (TNM) stage (*p* = 0.093) (Supplementary Table S4). These results were similar with the GSE76250 TNBC cohort.

Of the patients in the PUMCH TNBC cohort 1, 48.7% were older than 50 years, 50.3% had tumors larger than 2 cm, 43.3% had lymph node metastasis, and 22.4% had more than 4 lymph nodes involved. According to the American Joint Committee on Cancer (AJCC, the eighth edition) (Amin et al., 2017)^,^ 67.4% of the patients were diagnosed with stage II-III cancer. The median of ki-67 index was 55%. Moreover, 50.8% of patients had a ki-67 index of greater than 50%. A total of 73.8% of patients had tumors with high histological grades. Furthermore, 81.3% of patients received chemotherapy, while 24.6% of patients received radiotherapy (Supplementary Table S5). The high-CXCL9 group was strongly associated with smaller tumors (*p* = 0.022) and early tumors stage (*p* = 0.033). For these patients, there was no correlation between CXCL9 mRNA expression level and age (*p* = 0.713), ki-67 index (*p* = 0.511), or histological grading (*p* = 0.227). CXCL9 mRNA expression level also did not affect whether the patients received chemotherapy (*p* = 0.124) or radiotherapy (*p* = 0.789) (Table 1). Comparing the PUMCH and TCGA TNBC cohorts, the latter included older patients (*p* = 0.001), large tumors (*p* < 0.001), advanced TMN stages (*p* < 0.001), and higher levels of CXCL9 mRNA expression (Supplementary Table S6).

**TABLE 1:** The CXCL9 mRNA expression levels and clinicopathological features of the PUMCH TNBC cohort 1 (n = 187).

Parameters	CXCL9-high	CXCL9-low	*p*-value
**Age**			0.713
<50 years	47 (50.0)	49 (52,7)	
≥50 years	47 (50.0)	44 (47.3)	
**Tumor stage**			0.022
pT1	54 (57.4)	39 (41.9)	
pT2	39 (41.5)	47 (50.5)	
pT3	1 (1.1)	7 (7.6)	
**Lymph node stage**			0.542
pN0	58 (61.7)	48 (51.6)	
pN1	18 (19.1)	21 (22.6)	
pN2	10 (10.6)	12 (12.9)	
pN3	8 (8.6)	12 (12.9)	
**TNM stage**			0.033
I	39 (41.5)	22 (23.7)	
II	37 (39.4)	46 (49.5)	
III	18 (19.1)	25 (26.9)	
**Ki 67**			0.511
≤50	44 (46.8)	48 (51.6)	
>50	50 (53.2)	45 (48.4)	
**Histologic grade**			0.227
Well/Moderate	21 (22.3)	28 (30.1)	
Poor	73 (77.7)	65 (69.9)	
**Chemotherapy**			0.124
Yes	3 (3.2)	10 (10.8)	
No	79 (84.0)	73 (78.4)	
Unknown	12 (12.8)	10 (10.8)	
**Radiotherapy**			0.789
Yes	62 (66.0)	57 (61.3)	
No	22 (23.4)	24 (25.8)	
Unknown	10 (10.6)	12 (12.9)	

TNBC, triple-negative breast cancer; TNM, tumour-node-metastasis.

**FIGURE 2:**
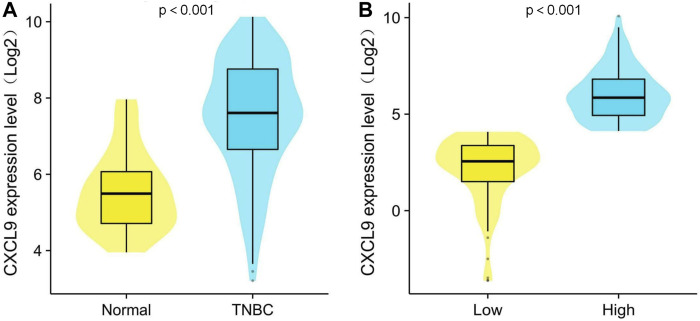
Comparison of CXCL9 mRNA expression level between **(A)** normal breast tissue and triple-negative breast cancer (TNBC) and **(B)** high- and low-CXCL9 groups in the PUMCH TNBC cohort 1.

In the GSE76250 cohort, the CXCL9 mRNA expression level was significantly higher in the TNBC group compared with normal groups (*p* < 0.001) (Figure 2A). In the PUMCH cohort 1, high-CXCL9 expression group had significantly higher level of CXCL9 than low group (*p* < 0.001) (Figure 2B).

### 3.2 CXCL9 mRNA expression level and survival in the TNBC cohorts

**FIGURE 3:**
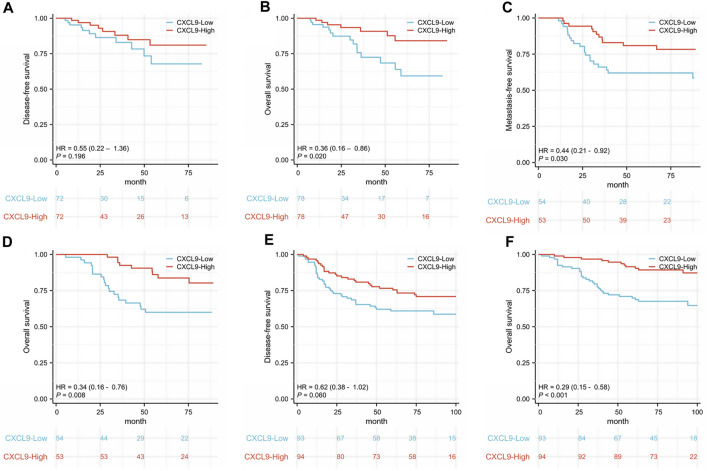
Kaplan-Meier survival analysis of CXCL9 mRNA in patients with triple-negative breast cancer (TNBC). **(A)** Disease-free survival (DFS) and **(B)** overall survival (OS) of the TCGA TNBC cohort. **(C)** Metastasis-free survival (MFS) and **(D)** OS of the GSE58812 cohort. **(E)** DFS and **(F)** OS of the PUMCH TNBC cohort 1.

In the TCGA TNBC cohort, there was no difference in DFS (*p* = 0.196, Figure 3A), while the high-CXCL9 group had better OS (*p* = 0.020, Figure 3B). The univariate Cox redgression analysis showed that high-CXCL9 correlated with better OS (Hazard ratio (HR) = 0.365, 95% Confidence interval (CI):0.156–0.855, *p* = 0.020). However, in the multivariate Cox regression analysis, a high CXCL9 level was not related to the outcome (HR = 0.437, 95% CI:0.179–1.070, *p* = 0.070, Supplementary Table S7). In the GSE58812 TNBC cohort, age at diagnosis of TNBC was from 28 to 85 years old, 31 patients occurred metastasis events and 29 patients dead. In this cohort, high-CXCL9 group had significantly prolonged MFS (*p* = 0.030, Figure 3C)and OS (*p* = 0.008, Figure 3D) compared with low group.

In the PUMCH TNBC cohort 1, there was no difference in DFS (*p* = 0.060, Figure 3E), while the high-CXCL9 group had prolonged OS (*p* < 0.001, Figure 3F) compared with the low-CXCL9 group. Univariate Cox regression analysis showed that CXCL9 could predict better OS outcomes (HR = 0.294, 95% CI:0.148–0.586, *p* = 0.001). However, in the multivariate Cox regression analysis, high CXCL9 did not independently predict the survival outcome (HR = 0.993, 95% CI:0.346–2.849, *p* = 0.99), when compared with age, T stage, N stage, ki-67 index, and histological grades (Table 2).

**TABLE 2:** The univariate and multivariate overall survival analyses for patients in the PUMCH TNBC cohort 1 (*n* = 187).

	Univariate analysis		Multivariate analysis	
	HR (95% CI)	*p*-value	HR (95% CI)	*p*-value
**Age** (<50/≥50)	1.141 (0.623–2.090)	0.670	0.941 (0.506–1.749)	0.848
**T** (1/2/3)	2.196 (1.301–3.706)	**0.003**	1.383 (0.792–2.415)	0.255
**N** (0/1/2/3)	2.086 (1.606–2.710)	**<0.001**	1.990 (1.502–2.636)	**<0.001**
**TNM** (I/II/III)	2.602 (1.669–4.056)	**<0.001**	—	—
**ki 67**(≤50%/>50%)	0.700 (0.380–1.291)	0.253	0.764 (0.405–1.442)	0.407
**Histologic grade** (I + II/III)	0.763 (0.396–1.468)	0.418	0.955 (0.473–1.926)	0.897
**CXCL9** (Low/High)	0.294 (0.148–0.586)	**0.001**	0.993 (0.346–2.849)	0.990

TNBC, triple-negative breast cancer; TNM, tumour-node-metastasis; CI, confidence interval.

### 3.3 The correlation of CXCL9 location, quantity, clinical features, and survival in the PUMCH TNBC Cohort2

**FIGURE 4:**
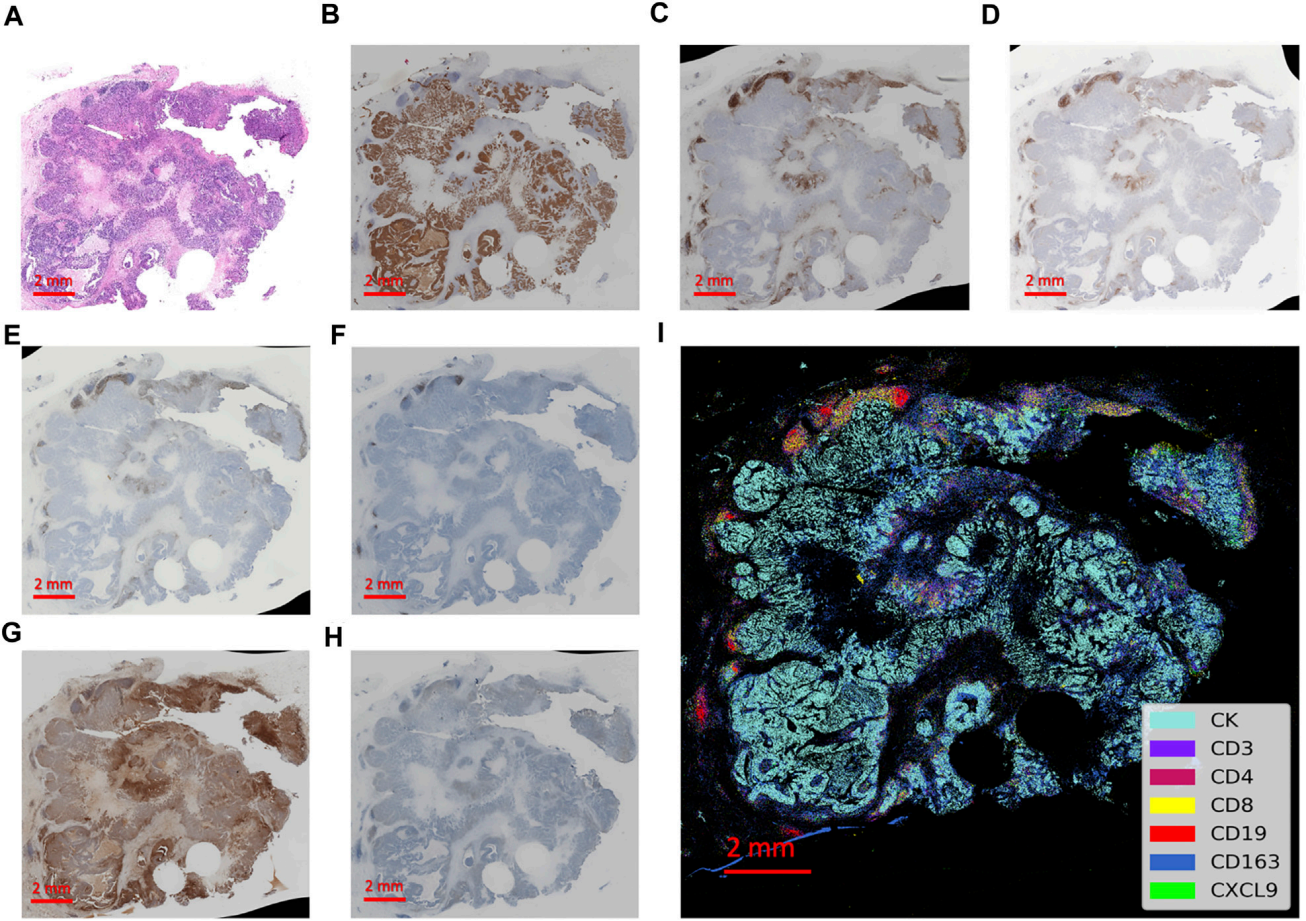
Integrated visualization of serial slides by computer-processed pseudo-coloring with every biomarker represented by a different color. **(A)** The hematoxylin-eosin staining of the tissue section. **(B–H)** The immunohistochemistry staining of immune markers of CK, CD3, CD4, CD8, CD19, CD163 and CXCL9, respectively. **(I)** The integrated visualization of the slides with partial zoomed-in details on the right.

A total of 69 patients in the PUMCH TNBC cohort 2 were analyzed for CXCL9 location and quantity in the TME of TNBC (Figure 4). In this cohort, 56.5% were older than 50 years, 68% of patients had tumors larger than 2 cm, 53.6% had lymph node metastasis, 30.4% had more than 4 lymph nodes involved, and 79.7% had stage II-III lymph node metastasis and 44.9% of patients demonstrated high histological grades (Supplementary Table S8).

The positive cell density and percentage of CXCL9 in immune cells in both TC and IM were calculated, as described above. ROC analyses were used to obtain the optimal cut-off value for the CXCL9 density and percentage measures at different locations for the prediction of OS (alive status). The areas under the curve (AUCs) were 0.784, 0.780, 0.797, 0.793, 0.779, and 0.802 for the density in TC, IM, both TC and IM, and percentage in TC, IM, and both TC and IM, respectively (all *p* < 0.001). The sensitivity ranged from 84% to 96%, while the specificity ranged from 61% to 72% (Supplementary Table S9).

Further analyses showed that CXCL9 was lowly expressed in patients with more lymph node metastasis according to density in both TC and IM (*p* = 0.018), percentage quantification in TC (*p* = 0.043), and percentage quantification in both TC and IM (*p* = 0.036). Moreover, CXCL9 was lowly expressed in tumors with high histological grade according to the percentage in IM (*p* = 0.031) (Table 3). Spearman correlation analyses showed that CXCL9 in TC strongly and positively correlated with CXCL9 in IM (r = 0.879, *p* < 0.001), and both TC and IM (r = 0.993, *p* < 0.001), while CXCL9 in IM also strongly and positively correlated with CXCL9 in TC and IM (r = 0.914, *p* < 0.001), according to the density. Similar results according to the percentage showed that CXCL9 expression was strongly and positively correlated with each other in TC, IM, as well as both TC and IM (all r > 0.800, *p* < 0.001) (Supplementary Table S10).

**TABLE 3:** The CXCL9 expression and clinicopathological features of the PUMCH TNBC cohort 2 (*n* = 69).

Parameters	D-TC high	D-TC low	*p*-value	D-IM high	D-IM low	*p*-value	D-TC + IM High	D-TC + IM Low	*p*-value	P-TC High	P-TC low	*p*-value	P-IM high	P-IM low	*p*-value	P-TC + IM High	P-TC + IM Low	*p*-value
**Age**			0.516			0.829			0.887			0.646			0.648			0.871
<50 years	21	9		24	6		22	8		20	10		23	7		21	9	
≥50 years	30	9		32	7		28	11		28	11		28	11		28	11	
**Tumour stage**			0.971			0.925			0.993			0.970			0.666			0.950
pT1	21	8		24	5		21	8		20	9		23	6		21	8	
pT2	27	9		29	7		26	10		25	11		25	11		25	11	
pT3	3	1		3	1		3	1		3	1		3	1		3	1	
**Lymph node**			0.069			0.100			0.018			0.043			0.135			0.036
pN0	26	6		27	5		26	6		24	8		25	7		24	8	
pN1	11	5		14	2		11	5		11	5		13	3		12	4	
pN2	9	1		9	1		9	1		9	1		8	2		9	1	
pN3	5	6		6	5		4	7		4	7		5	6		4	7	
**TNM stage**			0.744			0.471			0.522			0.762			0.413			0.624
I	11	3		12	2		11	3		10	4		11	3		10	4	
II	25	8		28	5		25	8		24	9		26	7		25	8	
III	15	7		16	6		14	8		14	8		14	8		14	8	
**Histologic grade**			0.108			0.051			0.061			0.177			0.031			0.10008
Well + Moderate	31	7		34	4		31	7		29	9		32	6		30	8	
Poor	20	11		22	9		19	12		19	12		19	12		19	12	

TNBC, triple-negative breast cancer; D, density; P, percentage; TC, tumour core; IM, invasive margin; TNM, tumour-node-metastasis.

**FIGURE 5:**
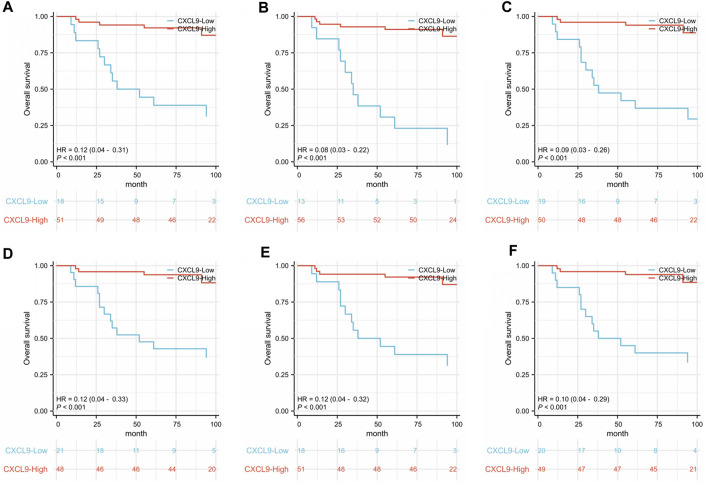
In the PUMCH triple-negative breast cancer cohort 2, correlations were detected between overall survival and **(A)** CXCL9 expression in the tumors core (TC); **(B)** CXCL9 expression in the invasive margin (IM); **(C)** CXCL9 expression in both the TC and IM according to the density; **(D)** CXCL9 expression in TC; **(E)** CXCL9 expression in IM; **(F)** CXCL9 expression in both TC and IM according to the percentage.

Survival analyses showed that high-CXCL9 expression was related to prolonged OS in TC (*p* < 0.001, Figure 5A), IM (*p* < 0.001, Figure 5B), as well as TC and IM (*p* < 0.001, Figure 5C) according to the density, and in TC (*p* < 0.001, Figure 5D), IM (*p* < 0.001, Figure 5E), as well as TC and IM (*p* < 0.001, Figure 5F) according to the percentage, compared with the low-CXCl9 group. Univariate analysis showed that high-CXCL9 expression was an independent factor for better outcomes. Multivariate Cox regression analyses of the PUMCH cohort data showed that, compared with age, T stage, N stage, ki 67 index and histological grading, high-CXCL9 expression, in any location according to both density (all *p* < 0.001, Table 4) and percentage(all *p* < 0.001, Table 5), could predict better OS.

**TABLE 4:** The uni- and multivariate overall survival analyses based on the CXCL9 density for patients in the PUMCH TNBC cohort 2 (*n* = 69).

	Univariate analysis		CXCL9 D-TC Multivariate analysis		CXCL9 D-IM Multivariate analysis		CXCL9 D-TC + IM Multivariate analysis	
	HR (95 CI)	*p*-value	HR (95 CI)	*p*-value	HR (95 CI)	*p*-value	HR (95 CI)	*p*-value
**Age** (<50/≥50)	1.143 (0.443–2.949)	0.783	1.093 (0.404–2.958)	0.860	0.970 (0.322–2.922)	0.957	0.980 (0.340–2.822)	0.970
**T** (1/2/3)	2.242 (1.036–4.854)	0.041	1.887 (0.772–4.612)	0.164	1.566 (0.657–3.730)	0.311	1.955 (0.796–4.966)	0.159
**N** (0/1/2/3)	1.678 (1.129–2.493)	0.010	1.263 (0.796–2.004)	0.321	1.242 (0.776–1.989)	0.367	1.161 (0.737–1.829)	0.521
**TNM** (I/II/III)	2.326 (1.121–4.827)	0.023	—	—	—	—	—	—
**ki 67**(≤50%/>50%)	0.494 (0.195–1.254)	0.138	1.051 (0.322–3.431)	0.934	1.134 (0.309–4.158)	0.849	0.766 (0.253–2.314)	0.636
**Histologic grade** ((Well + Moderate)/Poor)	2.405 (0.929–6.223)	0.070	1.411 (0.444–4.483)	0.560	1.328 (0.436–4.044)	0.617	1.453 (0.460–4.652)	0.519
**CXCL9 D-TC** (Low/High)	0.116 (0.043–0.312)	<0.001	0.111 (0.036–0.342)	<0.001	—	—	—	—
**CXCL9 D- IM** (Low/High)	0.082 (0.031–0.217)	<0.001	—	—	0.092 (0.030–0.284)	<0.001	—	—
**CXCL9 D- TC + IM** (Low/High)	0.091 (0.032–0.260)	<0.001	—	—	—	—	0.094 (0.031–0.290)	<0.001

TNBC, triple-negative breast cancer; D, density; TC, tumour core; IM, invasive margin; TNM, tumour-node-metastasis.

**TABLE 5:** The uni- and multivariate overall survival analyses based on the CXCL9 percentage for patients in the PUMCH TNBC cohort 2 (*n* = 69).

	Univariate analysis		CXCL9 P-TC Multivariate analysis		CXCL9 P-IM Multivariate analysis		CXCL9 P-TC + IM Multivariate analysis	
	HR (95 CI)	*p*-value	HR (95 CI)	*p*-value	HR (95 CI)	*p*-value	HR (95 CI)	*p*-value
**Age** (<50/≥50)	1.143 (0.443–2.949)	0.783	1.109 (0.395–3.115)	0.844	0.894 (0.286–2.794)	0.847	1.018 (0.363–2.858)	0.973
**T** (1/2/3)	2.242 (1.036–4.854)	0.041	1.860 (0.753–4.593)	0.179	1.538 (0.610–3.876)	0.362	1.736 (0.700–4.304)	0.234
**N** (0/1/2/3)	1.678 (1.129–2.493)	0.010	1.277 (0.813–2.005)	0.288	1.290 (0.799–2.085)	0.298	1.294 (0.829–2.020)	0.257
**TNM** (I/II/III)	2.326 (1.121–4.827)	0.023	—	—	—	—	—	—
**ki 67**(≤50%/>50%)	0.494 (0.195–1.254)	0.138	0.975 (0.307–3.093)	0.965	0.811 (0.244–2.701)	0.733	0.972 (0.315–2.998)	0.960
**Histologic grade** ((Well + Moderate)/Poor)	2.405 (0.929–6.223)	0.070	1.612 (0.492–5.285)	0.430	1.280 (0.394–4.159)	0.682	1.554 (0.482–5.013)	0.46
**CXCL9 P-TC** (Low/High)	0.116 (0.041–0.326)	<0.001	0.107 (0.034–0.336)	<0.001	—	—	—	—
**CXCL9 P-IM** (Low/High)	0.119 (0.044–0.320)	<0.001	—	—	0.148 (0.049–0.444)	0.001	—	—
**CXCL9 P-TC + IM** (Low/High)	0.103 (0.037–0.293)	<0.001	—	—	—	—	0.101 (0.032–0.316)	<0.001

TNBC, triple-negative breast cancer; P, percentage; TC, tumour core; IM, invasive margin; TNM, tumour-node-metastasis.

### 3.4 The correlation of CXCL9 expression and other immune markers in microenviroment

In the further Spearman’s correlation analysis, expression of CXCL9 in TC correlated with CD3 (ρ = 0.478, *p* < 0.001), CD4 (ρ = 0.485, *p* < 0.001), CD8 (ρ = 0.433, *p* < 0.001) and CD163 (ρ = 0.287, *p* = 0.017) according to density, expression of CXCL9 in IM correlated with CD3 (ρ = 0.509, *p* < 0.001), CD4 (ρ = 0.491, *p* < 0.001), CD8 (ρ = 0.412, *p* < 0.001) and CD163 (ρ = 0.357, *p* = 0.003) according to density, expression of CXCL9 in both TC and IM correlated with CD3 (ρ = 0.486, *p* < 0.001), CD4 (ρ = 0.499, *p* < 0.001), CD8 (ρ = 0.448, *p* < 0.001) and CD163 (ρ = 0.310, *p* = 0.010) according to density. Expression of CXCL9 in TC correlated with CD3 (ρ = 0.478, *p* < 0.001), CD4 (ρ = 0.456, *p* < 0.001) and CD8 (ρ = 0.271, *p* = 0.024) according to percentage, expression of CXCL9 in IM correlated with CD3 (ρ = 0.484, *p* < 0.001) and CD4 (ρ = 0.356, *p* = 0.003) according to percentage, expression of CXCL9 in both TC and IM correlated CD3 (ρ = 0.484, *p* < 0.001), CD4 (ρ = 0.441, *p* < 0.001), CD8 (ρ = 0.272, *p* = 0.024) and CD19 (ρ = 0.249, *p* = 0.040) according to percentage (Supplementary Table S11). Expression of CXCL9 in TC, in IM, and in both TC and IM according to densiry showed moderately correlation with expression of CD3 (ρ = 0.478–0.509, all *p* < 0.001) and moderately correlation with expression of CD4 (ρ = 0.485–0.499, all *p* < 0.001). Expression of CXCL9 in TC, in IM, and in both TC and IM according to percentage showed moderately correlation with expression of CD3 (ρ = 0.478–0.484, all *p* < 0.001). Expression of CXCL9 in TC and in both TC and IM according to percentage showed moderately correlation with expression of CD4 (ρ = 0.441–0.456, both *p* < 0.001).

## 4 Discussion

To gain insights into the potential application of CXCL9 in TNBC, we explored the relationship between CXCL9 mRNA expression, clinicopathological parameters, and prognosis in both our and public cohorts. We further explored the expression landscape of CXCL9 in the immune microenvironment and its relationship with clinicopathological features and prognosis in the TNBC.

Previous studies have demonstrated that serum CXCL9 is upregulated in patients with breast cancer compared to healthy controls or benign breast tumors (Ruiz-Garcia et al., 2010; Narita et al., 2016). Analyses among different breast cancer subtypes showed that CXCL9 mRNA levels in tumor tissues were much higher in the TNBC subtypes than in the luminal subtypes (Li et al., 2020; Liang et al., 2021). In this study, we analyzed CXCL9 mRNA expression and its effects in our PUMCH cohort, TCGA TNBC, GSE76250 and GSE58812 cohorts. We found that CXCL9 was upregulated in small tumors, which is consistent with previous research showing that CXCL9 was downregulated in large TNBC tumors (Lv et al., 2021). CXCL9 is also upregulated in the early stage of TNBC, which is consistent with previous research showing that it is highly expressed in patients with early breast cancer (Li et al., 2020). Survival analyses showed that high-CXCL9 mRNA expression was related to significantly prolonged overall survival in our cohort and the public cohorts. These results are consistent with studies that demonstrated the correlation between high-CXCL9 expression and better outcome in patients with whole breast cancer (Razis et al., 2020), the ER-negative cohort (Liang et al., 2021), and the TNBC subtype (Lv et al., 2021).

In our previous research (Cao et al., 2023), we explored the CXCL9 expression pattern in the TNBC tumor microarray (TMA), and found that CXCL9 was mainly expressed in immune cells, but rarely in tumor cells. High expression of CXCL9 in immune cells was significantly related to the high expression of PD-L1 and better prognosis. Considering that the TMA was prepared by using a hollow needle to select 1 mm diameter area in the tumor tissue, so it was difficult to reflect the overall landscape of the TNBC TME. In this study, we explored the landscape of CXCL9 in the hematoxylin-eosin staining slides of TNBC and differentiated the expression and location through pathological image reading via artificial intelligence and evaluated its relationship with clinicopathological parameters and prognosis. We analyzed CXCL9 in the TC, IM, and both TC and IM according to different measurement methods including the density and percentage. The above analyses demonstrated that a high-CXCL9 expression was significantly related to a prolonged OS in the TNBC cohort, according to the location and measurement method. The univariate and multivariate Cox regression analyses demonstrated that a high-CXCL9 expression in the TNBC immune microenvironment was an independent protective factor for prognosis.

Studies on cutaneous melanoma showed that the IHC of CXCL9 was significantly elevated in tumor tissues compared with normal controls, and high-CXCL9 mRNA level was related to a better OS compared with the low-CXCL9 group (Huang et al., 2020). However, cervical cancer studies had demonstrated that the IHC of CXCL9 was elevated in tumor tissues compared to normal tissues but did not correlate with the outcome (Kong et al., 2021). Ovarian cancer studies had shown that CXCL9 overexpression could result in T cell accumulation and prolonged survival, and thus, have important synergistic roles in ICB (Seitz et al., 2022). Gastric cancer research has shown that the IHC of CXCL9 was elevated in the stromal compartment of the tumor tissues compared to apparently normal tissue, which was not related to survival, while a high-CXCL9 mRNA expression was associated with better OS in patients with Epstein-Barr virus-related gastric cancer (Raja et al., 2017; Mu et al., 2022). Our research and previous studies have confirmed that high CXCL9 mRNA expression in TNBC was associated with better prognosis. The relationship between the expression pattern (quantity and location) of CXCL9 in the TNBC immune microenvironment and prognosis was first explored in this study. We found that high expression of CXCL9 in the TNBC TME, whether at the center of the tumor or at the front of the invasion, was associated with better prognosis.

Previous research on gastric cancer showed that the activation of the CXCL9/CXCR3 axis upregulated the expression of PD-L1 through the STAT and PI3K-Akt pathways (Zhang et al., 2018). Ovarian cancer studies have shown that CXCL9 could indirectly impact the upregulation of PD-L1 (Seitz et al., 2022). Research on TNBC has also revealed that CXCL9 may stimulate MHC-II activity through the JAK-STAT pathway and modify the TNBC immune microenvironment, which in turn, may affect ICB therapy (Wu et al., 2022). A study on non-small cell lung cancer demonstrated that CXCL9, a protein marker, could predict the prognosis of immunotherapy based on PD-1/PD-L1 blockade (Eltahir et al., 2021). Recent studies on gastric cancer and ovarian cancer have also shown that CXCL9 could enhance or reverse resistance to immunotherapy related to the PD-1/PD-L1 axis (Zhao et al., 2020; Seitz et al., 2022). These results confirmed the important role of CXCL9 in the tumor immune microenvironment and its potential synergy with PD-1/PD-L1 blockade immunotherapy. In our previous sutdy (Cao et al., 2023), we found that high expression of CXCL9 in immune cells strongly and positively correlated to high expression of PD-L1 in immune cell and better survival outcome in TNBC. In this study, we did not validate the relationship between CXCL9 and PD-L1, but we explored and found that high levels of CXCL9 mRNA were closely associated with better outcome in TNBC, and high expression of CXCL9 in the TC or IM of TNBC was associated with better overall survival. Therefore, we speculate that CXCL9 may become a potential synergistic factor for PD-1/PD-L1 immunotherapy in TNBC. We hope to explore the mechanism relationship between CXCL9 and PD-1/PD-L1 axis in TNBC in the future.

Image registration has been widely used in the field of medical images including focus localization, intraoperative navigation, and radiological diagnosis, but its application pathology has been limited to date. In this study, we used computational imaging analysis for the CXCL9 expression analysis based on IHC images (common in routine pathology tests and diagnoses). We used image registration to obtain the spatial positions of the scanning images to understand the expression pattern of CXCL9 in TNBC. We investigated the CXCL9 expression in different tumor regions including TC, IM, and both TC and IM. The results showed that the expression of CXCL9 had a strong and positive correlation in TC, IM, and TC + IM regions, regardless of the density or percentage. Furthermore, high-CXCL9 expression was an independent factor for better prognosis in the TNBC immune microenvironment. Compared with the traditional pathological slide reading method, computational image analysis has its advantages including consistent conditions, lowly subjective reading errors, and more comprehensive and more accurate data. In the future, we plan to use this technique to explore the panoramic expression of important immune markers represented by CXCL9 in the immune microenvironment of breast cancer.

In conclusion, this study revealed that high-CXCL9 expression in TNBC at both the transcriptional and protein levels is related to better prognosis. In the immune microenvironment, high expression of CXCL9 in the tumor core or/and invasive margin were closely related to and should be considered as independent protective factors of prognosis. We also successfully applied computational image analysis in this study. In the future, its application is expected to extend to the exploration of the panoramic expression of the breast cancer immune microenvironment.

## Figures and Tables

**Table:** 

**TNBC**	triple-negative breast cancer
**ER**	estrogen receptor
**PR**	progesterone receptor
**HER-2**	human epidermal growth factor receptor-2
**PD-L1**	programmed cell death ligand 1
**PD-1**	programmed cell death
**ICB**	immune checkpoint blockade
**TME**	tumor microenvironment
**CTLs**	cytotoxic lymphocytes
**NK**	natural killer
**Th1**	T-helper 1
**IFN-γ**	interferon-γ
**PUMCH**	Peking Union Medical College Hospital
**FFPE**	formalin-fixed paraffin-embedded
**CPM**	counts per million
**H&E**	hematoxylin-eosin
**TC**	tumor core
**IF**	invasive front
**IM**	invasive margin
**BRCA**	breast invasive cancer cohort
**GEO**	Gene Expression Omnibus
**TCGA**	the Cancer Genome Atlas
**IHC**	immunohistochemical
**TMA**	tumor microarray
**ROC**	receiver operating curve
**DFS**	disease-free survival
**MFS**	metastasis-free survival
**OS**	overall survival
**TNM**	tumour-node-metastasis
**AJCC**	American Joint Committee on Cancer
**AUCs**	areas under the curve
**CI**	confidence interval
**HR**	hazard ratio

## Data Availability

The original contributions presented in the study are included in the article/Supplementary Material, further inquiries can be directed to the corresponding authors.
