# Supplementary figures and images for: Quantifying spatial CXCL9 distribution with image analysis predicts improved prognosis of triple-negative breast cancer
Source: Front Genet. 2024 Jun 18;15:1421573. doi: 10.3389/fgene.2024.1421573 (PMC11217326; doi:10.3389/fgene.2024.1421573)

E

CD8

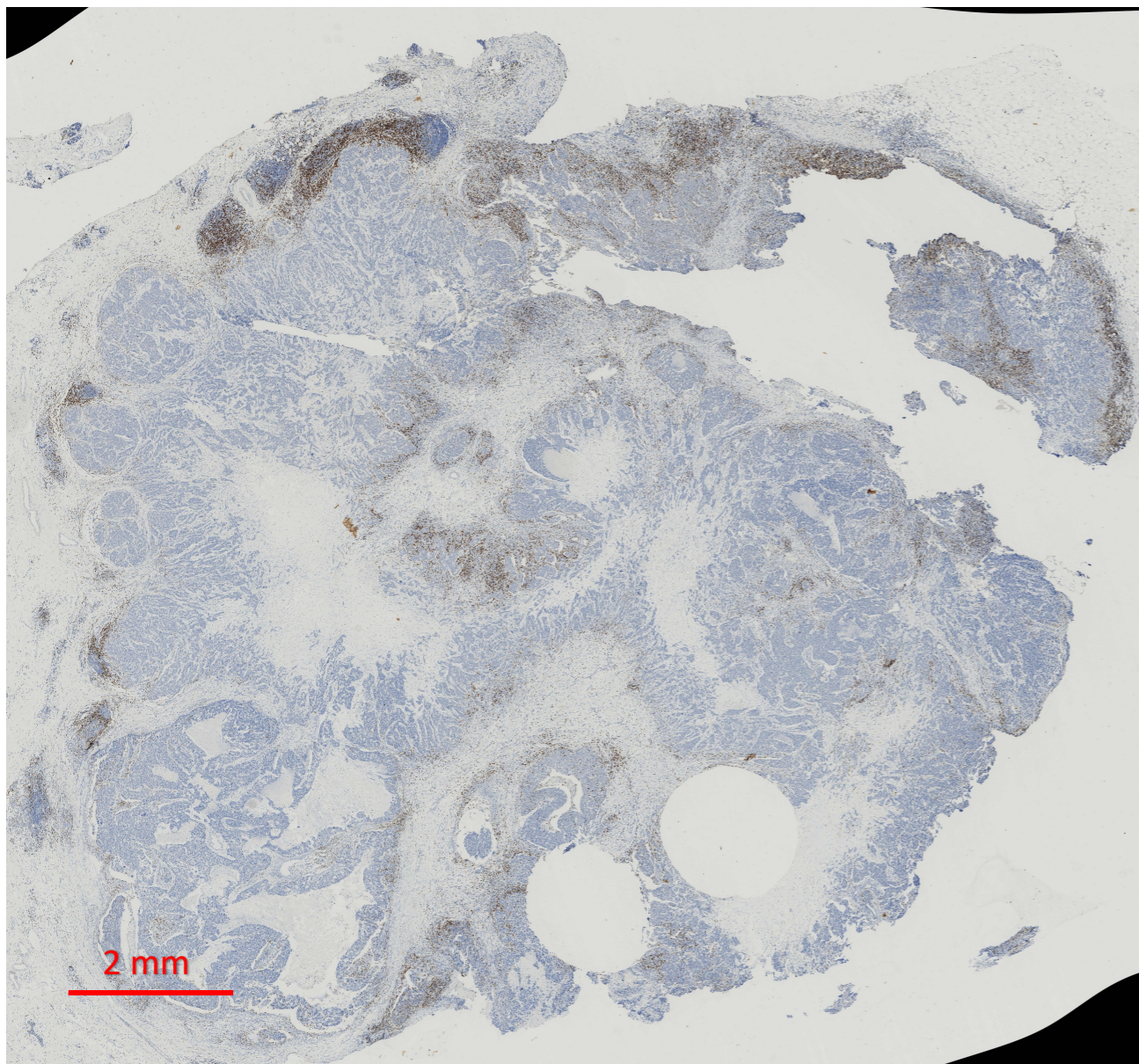

F

CD19

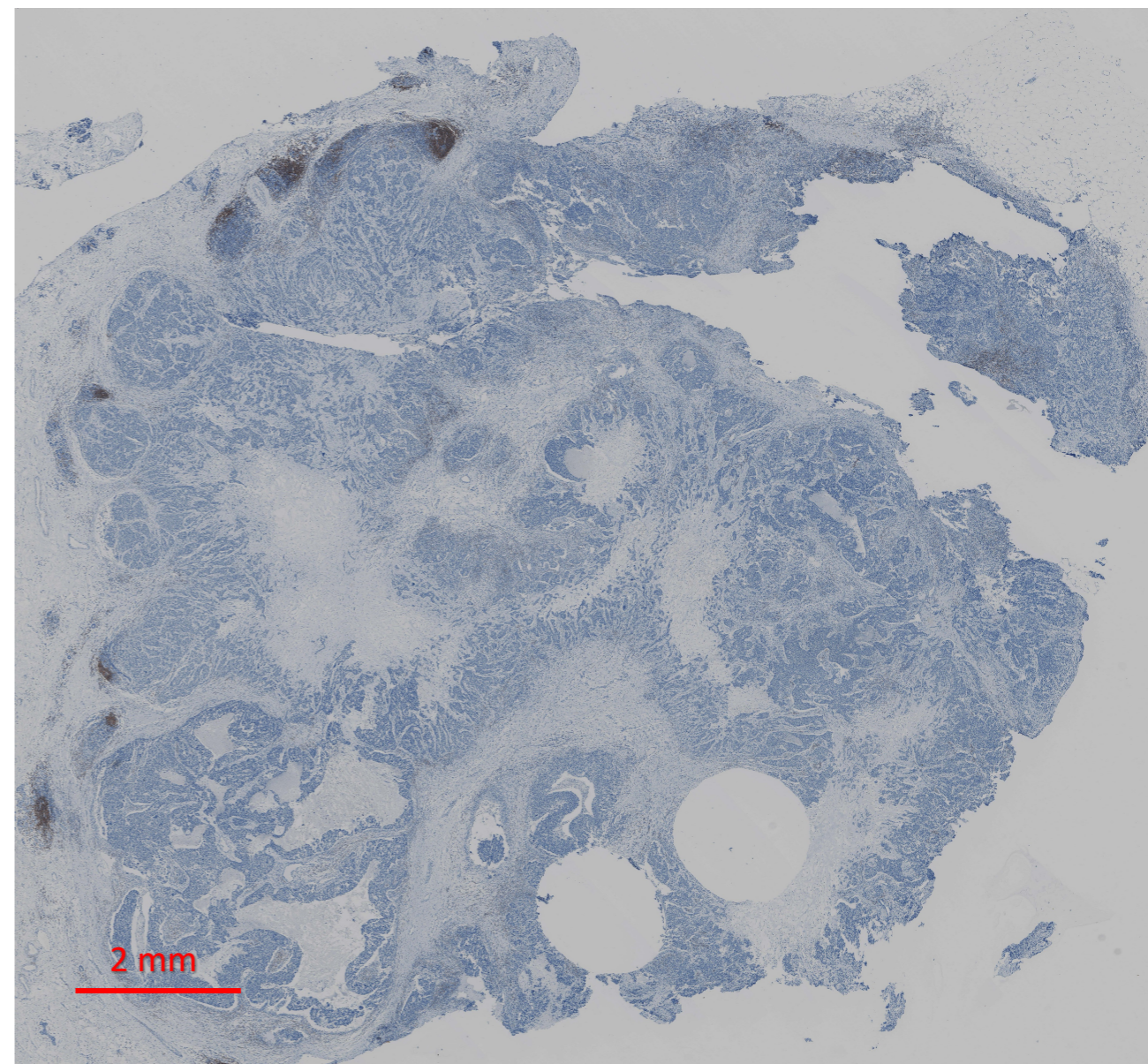

G

CD163

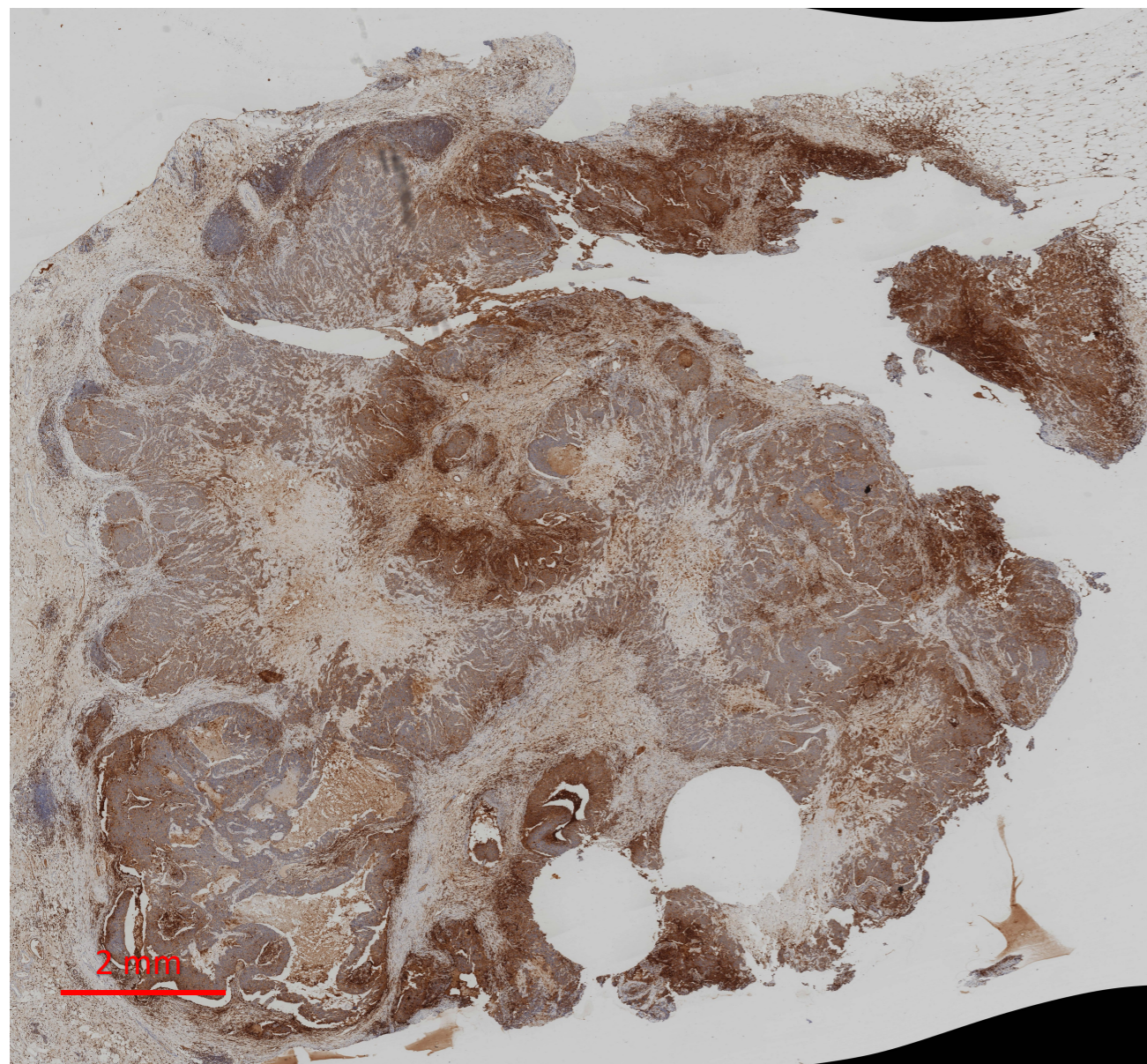

H

CXCL9

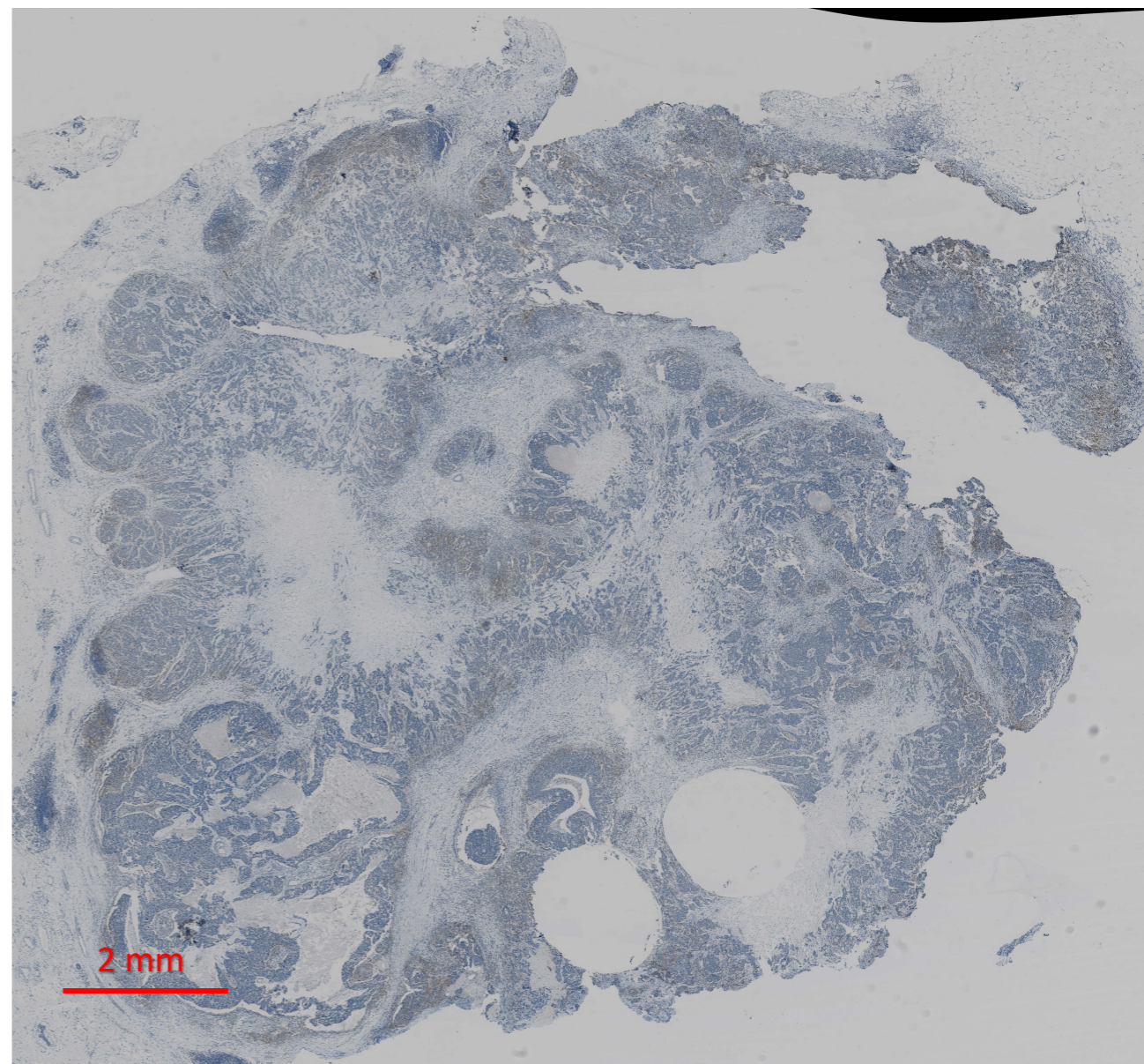

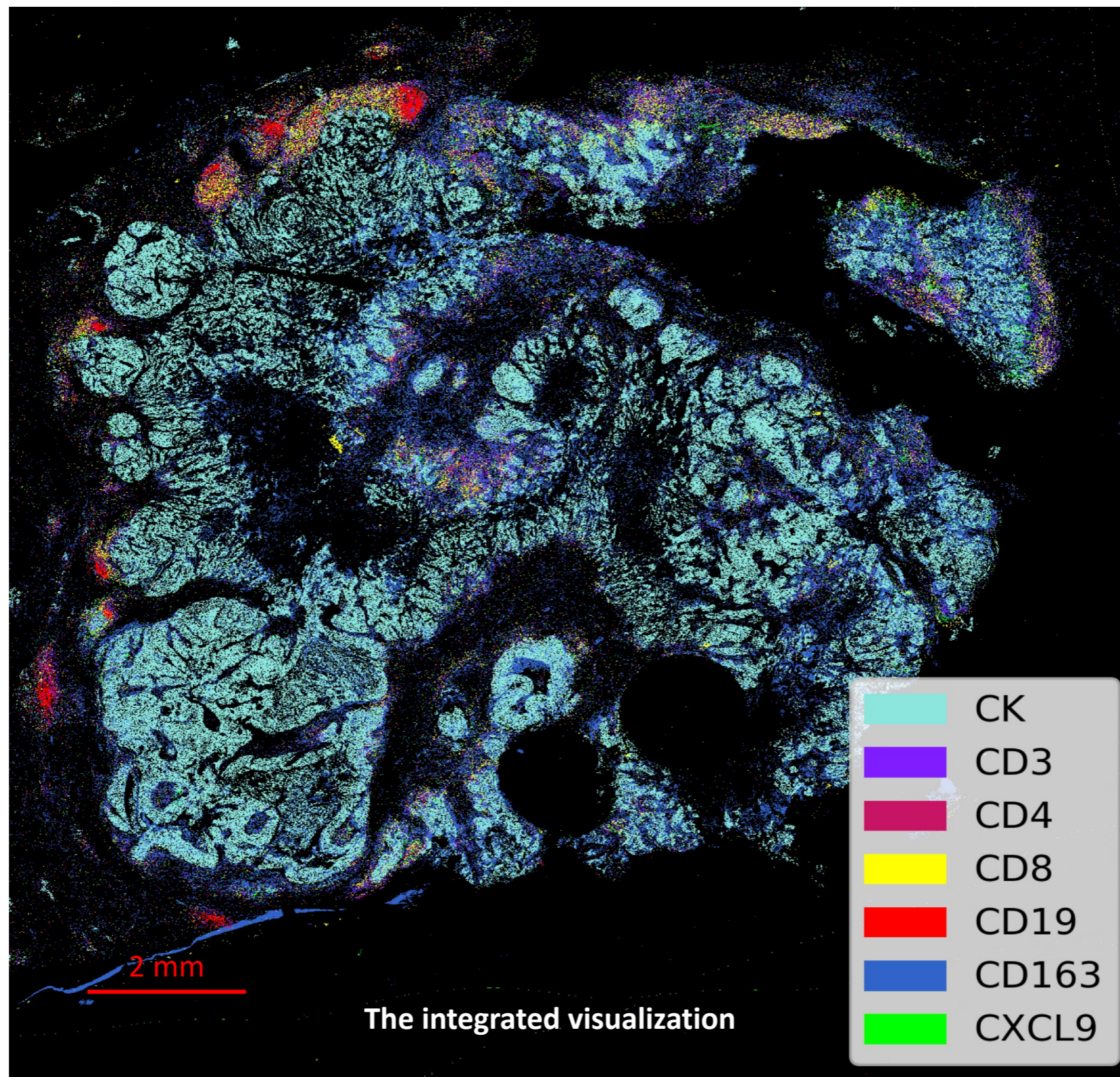

Supplement: Supplementary file 1 [file DataSheet2.PDF]

A

HE

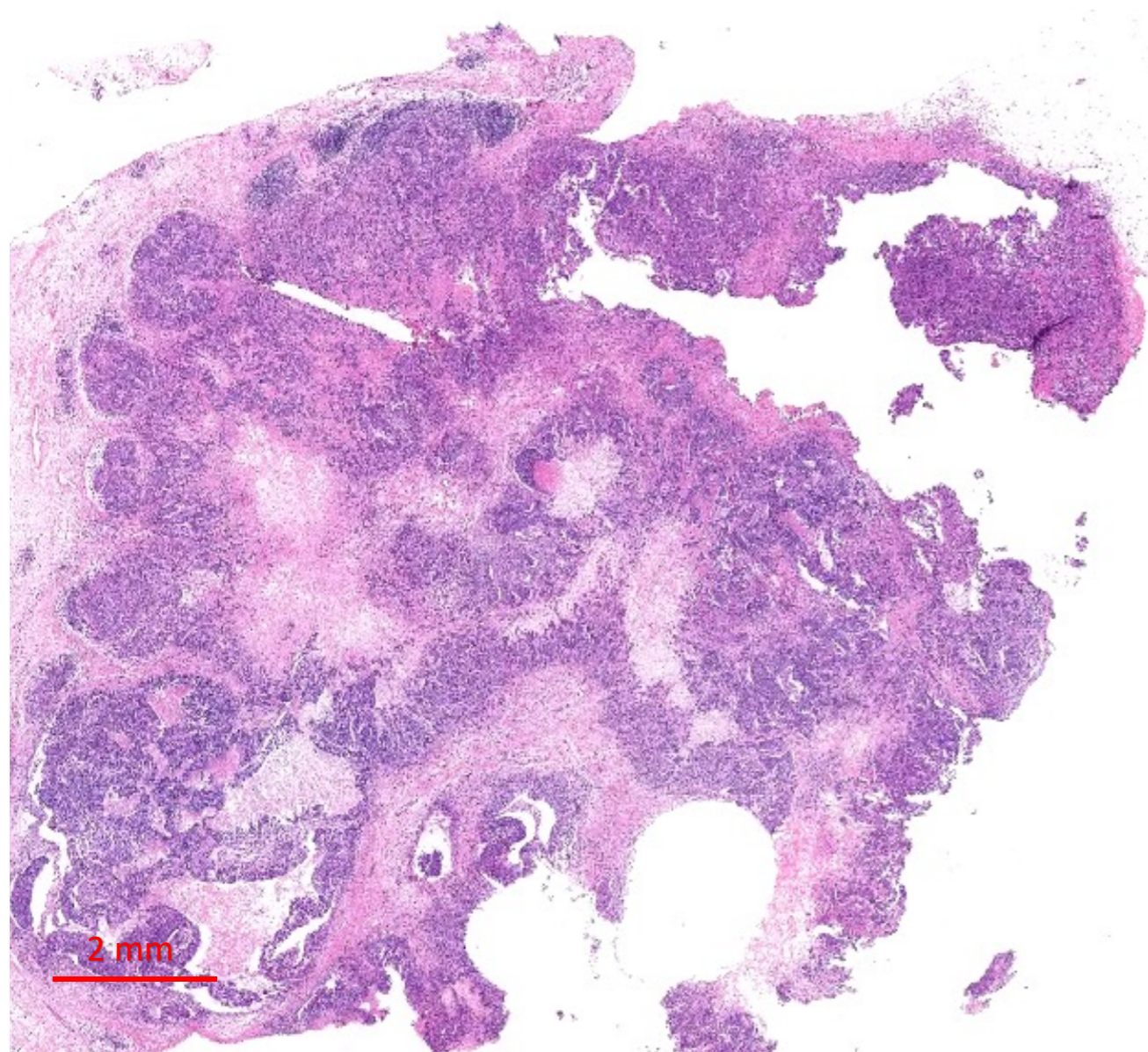

B

CK

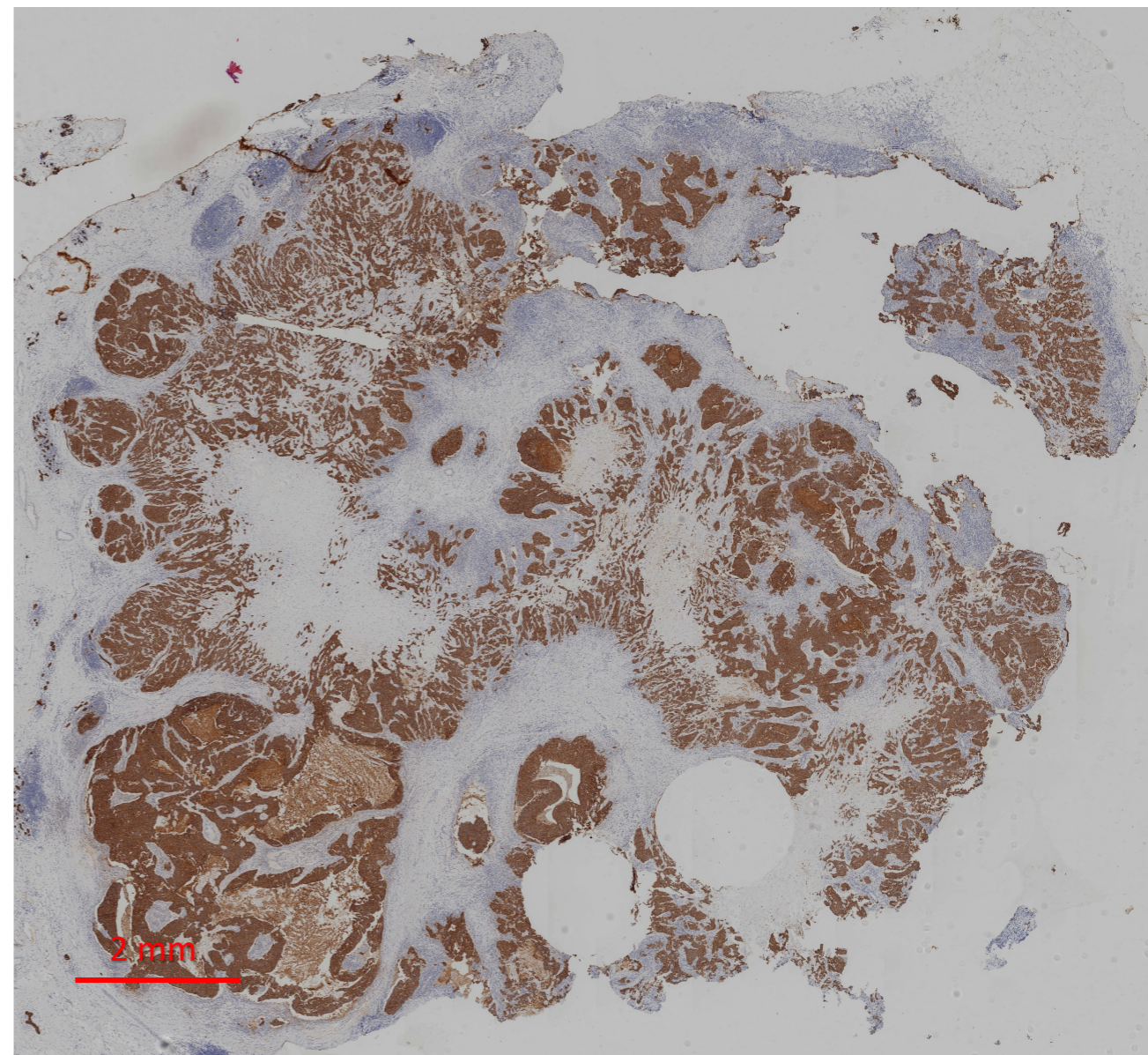

C

CD3

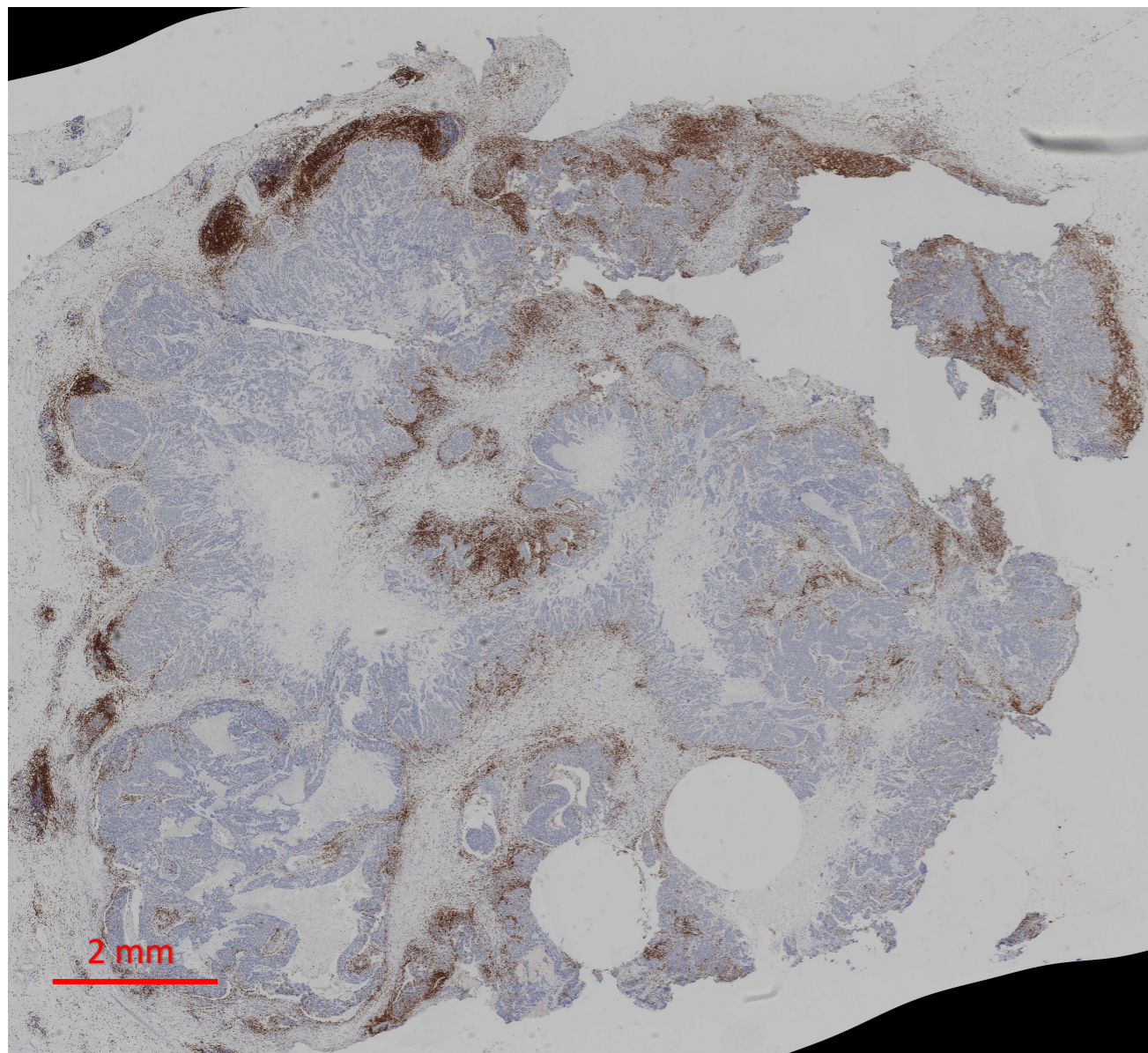

D

CD4

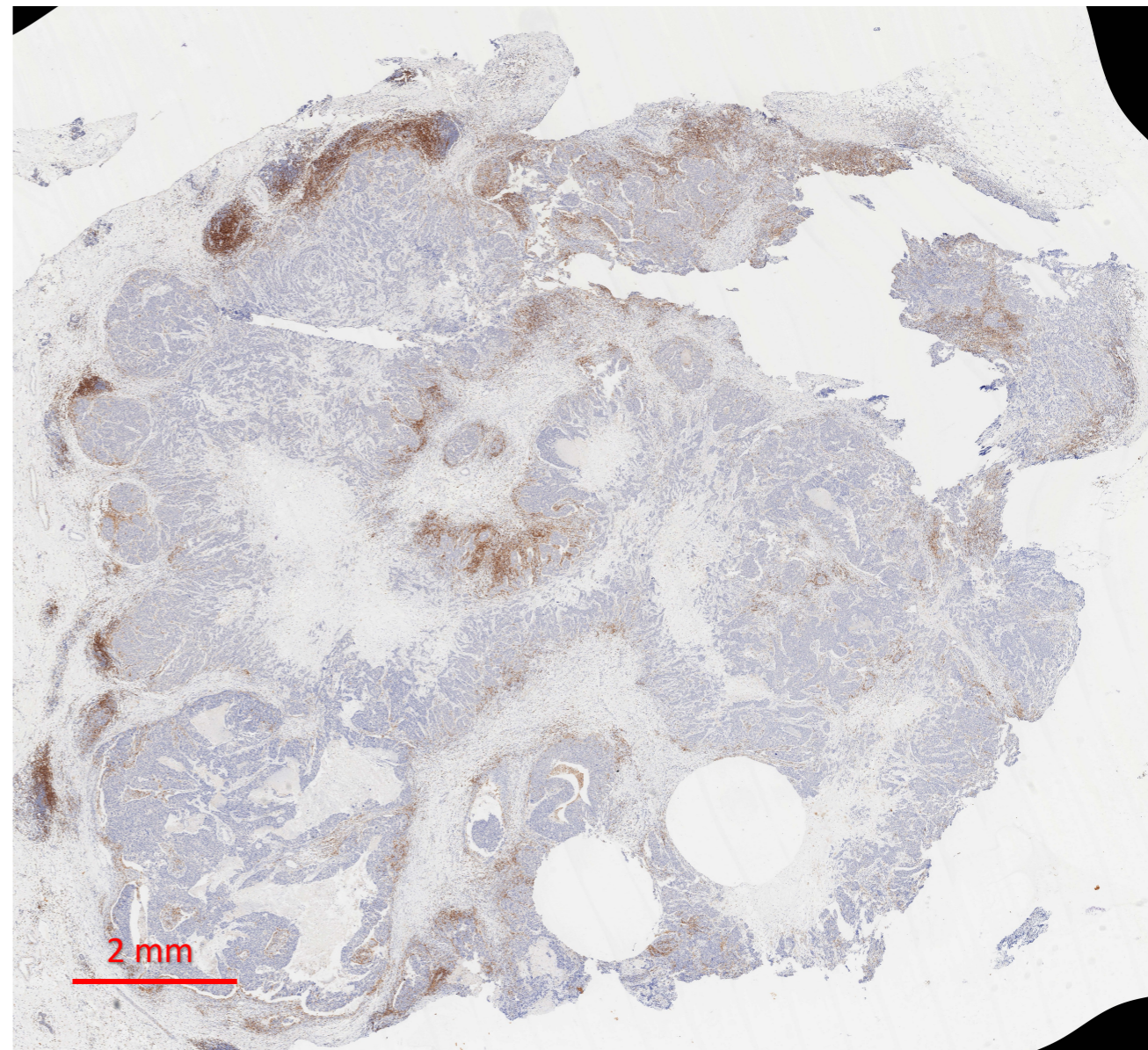

Supplement: Supplementary file 3 [file DataSheet1.PDF]
