# Supplementary material for: Quantifying spatial CXCL9 distribution with image analysis predicts improved prognosis of triple-negative breast cancer
Source: Front Genet. 2024 Jun 18;15:1421573. doi: 10.3389/fgene.2024.1421573 (PMC11217326; doi:10.3389/fgene.2024.1421573)
Supplement: Supplementary file 2 [file DataSheet3.ZIP › Supplementary Table 1_R1.docx]

**Supplementary Table 1.** Baseline of the GSE76250 cohort (n=154).

| **Characteristic** | **Value** |
| --- | --- |
|  |  |
| **Age, years (median, range)** | 54 (27-83) |
| **Age, n (%)** |  |
| <50 years | 55 (35.7) |
| ≥50 years | 99 (64.3) |
| **Tumor stage, n (%)** |  |
|  |  |
| pT1 | 57 (37.0) |
| pT2 | 94 (61.0) |
| pT3 | 3 (2.0) |
| **Lymph node stage, n (%)** |  |
|  |  |
| pN0 | 86 (55.8) |
| pN1 | 37 (24.0) |
| pN2 | 15(9.7) |
| pN3 | 16 (10.5) |
| **TNM stage, n (%)** |  |
|  |  |
| I | 36 (23.4) |
| II | 87 (56.5) |
| III | 31 (20.1) |
| **Menopause status, n (%)** |  |
| premenopausal | 60 (39.0) |
| postmenopausal | 94 (61.0) |
| **Ki 67, n (%)** |  |
| ≤50 | 72(46.8) |
| ＞50 | 82(53.2) |
| **Histologic grade** |  |
| Well/Moderate | 54(35.1) |
| Poor | 100(64.9) |

TNM, tumour-node-metastasis.
