# Supplementary material for: Quantifying spatial CXCL9 distribution with image analysis predicts improved prognosis of triple-negative breast cancer
Source: Front Genet. 2024 Jun 18;15:1421573. doi: 10.3389/fgene.2024.1421573 (PMC11217326; doi:10.3389/fgene.2024.1421573)
Supplement: Supplementary file 2 [file DataSheet3.ZIP › Supplementary Table 2_R1.docx]

**Supplementary Table 2.** The CXCL9 mRNA expression levels and clinicopathological features in the GSE76250 TNBC cohort (n=154).

| Parameters | CXCL9-High | CXCL9-Low | p value |
| --- | --- | --- | --- |
| **Age** |  |  | 0.130 |
| <50 years | 23(29.9) | 32(41.6) |  |
| ≥50 years | 54(70.1) | 45(58.4) |  |
| **Tumour stage** |  |  | 0.089 |
| pT1 | 35(45.5) | 22(28.6) |  |
| pT2 | 41(53.2) | 53(68.8) |  |
| pT3 | 1(1.3) | 2(2.6) |  |
| **Lymph node** |  |  | 0.442 |
| pN0 | 38(49.4) | 48(62.3) |  |
| pN1 | 21(27.2) | 16(20.8) |  |
| pN2 | 9(11.7) | 6(7.8) |  |
| pN3 | 9(11.7) | 7(9.1) |  |
| **TNM stage** |  |  | 0.477 |
| I | 19(24.7) | 17(22.1) |  |
| II | 40(51.9) | 47(61.0) |  |
| III | 18(23.4) | 13(16.9) |  |
| **Menopause status** |  |  | 0.186 |
| premenopausal | 26(33.8) | 34(44.2) |  |
| postmenopausal | 51(66.2) | 43(55.8) |  |
| **Ki 67** |  |  | 0.518 |
| ≤50 | 34(44.2) | 38(49.4) |  |
| ＞50 | 43(55.8) | 39(50.6) |  |
| **Histologic grade** |  |  | 0.927 |
| Well/Moderate | 26(33.8) | 28(36.4) |  |
| Poor | 51(66.2) | 49(63.6) |  |

TNBC, triple-negative breast cancer; TNM, tumour-node-metastasis.
