# Supplementary material for: Quantifying spatial CXCL9 distribution with image analysis predicts improved prognosis of triple-negative breast cancer
Source: Front Genet. 2024 Jun 18;15:1421573. doi: 10.3389/fgene.2024.1421573 (PMC11217326; doi:10.3389/fgene.2024.1421573)
Supplement: Supplementary file 2 [file DataSheet3.ZIP › Supplementary Table 3_R1.docx]

| **Characteristic** | **Value** |
| --- | --- |
|  |  |
| **Age, years (median, range)** | 55 (29-90) |
|  |  |
| **Age, n (%)** |  |
| <50 years | 51 (32.7) |
| ≥50 years | 105 (67.3) |
| **Tumor stage, n (%)** |  |
|  |  |
| pT1 | 40 (25.6) |
| pT2 | 99 (63.5) |
| pT3 | 13 (8.3) |
| pT4 | 4 (2.6) |
| **Lymph node stage, n (%)** |  |
|  |  |
| pN0 | 105 (67.3) |
| pN1 | 32 (20.5) |
| pN2 | 12(7.7) |
| pN3 | 7 (4.5) |
| **TNM stage, n (%)** |  |
|  |  |
| I | 28 (18.0) |
| II | 103 (66.0) |
| III | 25 (16.0) |

**Supplementary Table 3.** Baseline of the TCGA TNBC cohort (n=156).

TCGA, The Cancer Genome Atlas; TNBC, triple-negative breast cancer; TNM, tumour-node-metastasis; OS, overall survival.
