# Supplementary material for: Quantifying spatial CXCL9 distribution with image analysis predicts improved prognosis of triple-negative breast cancer
Source: Front Genet. 2024 Jun 18;15:1421573. doi: 10.3389/fgene.2024.1421573 (PMC11217326; doi:10.3389/fgene.2024.1421573)
Supplement: Supplementary file 2 [file DataSheet3.ZIP › Supplementary Table 4_R1.docx]

**Supplementary Table 4.** The CXCL9 mRNA expression levels and clinicopathological features in the TCGA TNBC cohort (n=156).

| Parameters | CXCL9-High | CXCL9-Low | p value |
| --- | --- | --- | --- |
| **Age** |  |  | 0.953 |
| <50 years | 26 (32.9) | 25 (32.5) |  |
| ≥50 years | 53 (67.1) | 52 (67.5) |  |
| **Tumour stage** |  |  | 0.150 |
| pT1 | 22 (27.8) | 18 (23.4) |  |
| pT2 | 52 (65.8) | 47 (61.0) |  |
| pT3 | 5 (6.4) | 8 (10.4) |  |
| pT4 | 0 (0.0) | 4 (5.2) |  |
| **Lymph node** |  |  | 0.096 |
| pN0 | 54 (68.4) | 51 (66.2) |  |
| pN1 | 20 (25.3) | 12 (15.6) |  |
| pN2 | 3 (3.8) | 9 (11.7) |  |
| pN3 | 2 (2.5) | 5 (6.5) |  |
| **TNM stage** |  |  | 0.093 |
| I | 17 (21.5) | 11 (14.3) |  |
| II | 54 (68.4) | 49 (63.6) |  |
| III | 8 (10.1) | 17 (22.1) |  |

TCGA, The Cancer Genome Atlas; TNBC, triple-negative breast cancer; TNM, tumour-node-metastasis.
