# Supplementary material for: Quantifying spatial CXCL9 distribution with image analysis predicts improved prognosis of triple-negative breast cancer
Source: Front Genet. 2024 Jun 18;15:1421573. doi: 10.3389/fgene.2024.1421573 (PMC11217326; doi:10.3389/fgene.2024.1421573)
Supplement: Supplementary file 2 [file DataSheet3.ZIP › Supplementary Table 5_R1.docx]

| **Characteristic** | **Value** |
| --- | --- |
|  |  |
| **Age, years (median, range)** | 49,25-80 |
|  |  |
| **Age, n (%)** |  |
| <50 years | 96 (51.3) |
| ≥50 years | 91 (48.7) |
| **Tumor stage, n (%)** |  |
|  |  |
| pT1 | 93 (49.7) |
| pT2 | 86 (46.0) |
| pT3 | 8 (4.3) |
| **Lymph node stage, n (%)** |  |
|  |  |
| pN0 | 106 (56.7) |
| pN1 | 39 (20.9) |
| pN2 | 22(11.8) |
| pN3 | 20 (10.6) |
| **TNM stage, n (%)** |  |
|  |  |
| I | 61(32.6) |
| II | 83 (44.4) |
| III | 43 (23.0) |
| **Ki 67 (median, range)** | 55, 5-95 |
| **Ki 67, n (%)** |  |
| ≤50 | 92 (49.2) |
| ＞50 | 95 (50.8) |
| **Histologic grade, n (%)** |  |
|  |  |
| Well | 5(2.7) |
| Moderate | 44(23.5) |
| Poor | 138 (73.8) |
| **Chemotherapy, n (%)** |  |
| Yes | 152 (81.3) |
| No | 13 (7.0) |
| Unknown | 22 (11.7) |
| **Radiotherapy, n (%)** |  |
| Yes | 46 (24.6) |
| No | 119 (63.6) |
| Unknown | 22 (11.8) |

**Supplementary Table 5.** Baseline of the PUMCH TNBC cohort 1 (n=187)

TNBC, triple-negative breast cancer; TNM, tumour-node-metastasis.
