# Supplementary material for: Quantifying spatial CXCL9 distribution with image analysis predicts improved prognosis of triple-negative breast cancer
Source: Front Genet. 2024 Jun 18;15:1421573. doi: 10.3389/fgene.2024.1421573 (PMC11217326; doi:10.3389/fgene.2024.1421573)
Supplement: Supplementary file 2 [file DataSheet3.ZIP › Supplementary Table 6_R1.docx]

**Supplementary Table 6.** Comparison between the baseline data of the PUMCH TNBC cohort 1 (n=187) and the TCGA TNBC cohort (n=156).

| **Characteristics** | **PUMCH (n=187)** | **TCGA (n=156)** | **p** |
| --- | --- | --- | --- |
| **Age, years (mean±SD)** | 50±12 | 55±12 | **＜0.001** |
| **Age, n (%)** |  |  | **0.001** |
| <50 years | 96 (51.3) | 51 (32.7) |  |
| ≥50 years | 91 (48.7) | 105 (67.3) |  |
| **Tumor stage, n (%)** |  |  | **＜0.001** |
| pT1 | 93 (49.7) | 40 (25.6) |  |
| pT2 | 86 (46.0) | 99 (63.5) |  |
| pT3 | 8 (4.3) | 13 (8.3) |  |
| pT4 | 0(0.0) | 4 (2.6) |  |
| **Lymph node, n (%)** |  |  | 0.067 |
| pN0 | 106 (56.7) | 105 (67.3) |  |
| pN1 | 39 (20.9) | 32 (20.5) |  |
| pN2 | 22 (11.8) | 12 (7.7) |  |
| pN3 | 20 (10.6) | 7 (4.5) |  |
| **TNM stage, n (%)** |  |  | **＜0.001** |
| I | 61 (32.6) | 28 (18.0) |  |
| II | 83 (44.4) | 103 (66.0) |  |
| III | 43 (23.0) | 25 (16.0) |  |
| **OS status, n (%)** |  |  | 0.098 |
| Alive | 145 (77.5) | 132 (84.6) |  |
| Dead | 42 (22.5) | 24 (15.4) |  |
| **CXCL9 mRNA**  (mean ± SD) | 4.1±2.5 | 6.7±2.4 | **＜0.001** |

TCGA, The Cancer Genome Atlas; TNBC, triple-negative breast cancer; TNM, tumour-node-metastasis; OS, overall survival.
