# Supplementary material for: Quantifying spatial CXCL9 distribution with image analysis predicts improved prognosis of triple-negative breast cancer
Source: Front Genet. 2024 Jun 18;15:1421573. doi: 10.3389/fgene.2024.1421573 (PMC11217326; doi:10.3389/fgene.2024.1421573)
Supplement: Supplementary file 2 [file DataSheet3.ZIP › Supplementary Table 7.docx]

**Supplementary Table 7.** The univariate and multivariate overall survival analyses of patients in the TCGA TNBC cohort (n=156).

|  | **Univariate analysis** | | **Multivariate analysis** | |
| --- | --- | --- | --- | --- |
|  | HR (95% CI) | p value | HR (95% CI) | p value |
| **Age** (<50/≥50) | 0.809 (0.362-1.810) | 0.606 | 0.561 (0.236-1.332) | 0.190 |
| **T** (1/2/3/4） | 2.676 (1.504-4.760) | **0.001** | 2.062 (1.049-4.053) | **0.036** |
| **N** (0/1/2/3） | 2.732 (1.826-4.060) | **＜0.001** | 2.586 (1.705-3.922) | **＜0.001** |
| **TNM** (I/II/III) | 6.983 (3.208-15.201) | **＜0.001** | — | — |
| **CXCL9** (Low/High) | 0.365 (0.156-0.855) | **0.020** | 0.437 (0.179-1.070) | 0.070 |

TNBC, triple-negative breast cancer; TNM, tumour-node-metastasis.
