# Supplementary material for: Quantifying spatial CXCL9 distribution with image analysis predicts improved prognosis of triple-negative breast cancer
Source: Front Genet. 2024 Jun 18;15:1421573. doi: 10.3389/fgene.2024.1421573 (PMC11217326; doi:10.3389/fgene.2024.1421573)
Supplement: Supplementary file 2 [file DataSheet3.ZIP › Supplementary Table 8.docx]

**Supplementary Table 8.** Baseline of the PUMCH TNBC cohort 2 (n=69).

| **Characteristic** | **Value** |
| --- | --- |
|  |  |
| **Age, n (%)** |  |
| <50 years | 30 (43.5) |
| ≥50 years | 39 (56.5) |
| **Tumor stage, n (%)** |  |
|  |  |
| pT1 | 29 (42.0) |
| pT2 | 36 (52.2) |
| pT3 | 4 (5.8) |
| **Lymph node, n (%)** |  |
|  |  |
| pN0 | 32 (46.4) |
| pN1 | 16 (23.2) |
| pN2 | 10 (14.5) |
| pN3 | 11 (15.9) |
| **TNM stage, n (%)** |  |
|  |  |
| I | 14 (20.3) |
| II | 33 (47.8) |
| III | 22 (31.9) |
| **Histologic grade, n (%)** |  |
|  |  |
| Well | 11 (15.9) |
| Moderate | 27 (39.2) |
| Poor | 31 (44.9) |

TNBC, triple-negative breast cancer; TNM, tumour-node-metastasis.
