# Supplementary material for: Quantifying spatial CXCL9 distribution with image analysis predicts improved prognosis of triple-negative breast cancer
Source: Front Genet. 2024 Jun 18;15:1421573. doi: 10.3389/fgene.2024.1421573 (PMC11217326; doi:10.3389/fgene.2024.1421573)
Supplement: Supplementary file 2 [file DataSheet3.ZIP › Supplementary Table 9.docx]

**Supplementary Table 9**. Receiver operating curve analyses based on CXCL9 density and percentage at different location for the prediction of overall survival (alive status) in the PUMCH TNBC cohort 2 (n=69).

| Parameter | Site | AUC | p value | Sensitivity | Specificity |
| --- | --- | --- | --- | --- | --- |
| Density | Tumor core | 0.784 | ＜0.001 | 88% | 67% |
|  | Invasive margin | 0.780 | ＜0.001 | 96% | 61% |
|  | Both | 0.797 | ＜0.001 | 88% | 72% |
| Percentage | Tumor core | 0.793 | ＜0.001 | 84% | 72% |
|  | Invasive margin | 0.779 | ＜0.001 | 88% | 67% |
|  | Both | 0.802 | ＜0.001 | 86% | 72% |
