# Supplementary material for: Quantifying spatial CXCL9 distribution with image analysis predicts improved prognosis of triple-negative breast cancer
Source: Front Genet. 2024 Jun 18;15:1421573. doi: 10.3389/fgene.2024.1421573 (PMC11217326; doi:10.3389/fgene.2024.1421573)
Supplement: Supplementary file 2 [file DataSheet3.ZIP › Supplementary Table 10.docx]

**Supplementary Table 10.** Spearman correlation analyses between CXCL9 expression level and location in the PUMCH TNBC cohort 2 (n=69).

|  | **According to Density** | | |  | **According to Percentage** | | |
| --- | --- | --- | --- | --- | --- | --- | --- |
| **CXCL9** | TC | IM | TC+IM | **CXCL9** | TC | IM | TC+IM |
| TC | 1.000 | - | - | TC | 1.000 | - | - |
| IM | **0.879** | 1.000 | - | IM | **0.858** | 1.000 | - |
| TC+IM | **0.993** | **0.914** | 1.000 | TC+IM | **0.99** | **0.896** | 1.000 |

TNBC, triple-negative breast cancer; D, density; P, percentage; TC, tumour core; IM, invasive margin.
